# Combined Effects of UV-B and Drought on Native and Exotic Populations of *Verbascum thapsus* L.

**DOI:** 10.3390/plants9020269

**Published:** 2020-02-18

**Authors:** Maria Hock, Carolin Plos, Maria Sporbert, Alexandra Erfmeier

**Affiliations:** 1 Kiel University, Institute for Ecosystem Research/Geobotany, Olshausenstr. 75, 24118 Kiel, Germany; aerfmeier@ecology.uni-kiel.de; 2 Martin Luther University Halle-Wittenberg, Institute of Biology/Geobotany and Botanical Garden, Am Kirchtor 1, 06108 Halle, Germany; carolin.plos@botanik.uni-halle.de (C.P.); maria.sporbert@botanik.uni-halle.de (M.S.); 3 German Centre for Integrative Biodiversity Research (iDiv) Halle-Jena-Leipzig, Deutscher Platz 5E, 04103 Leipzig, Germany; 4 Friedrich Schiller University Jena, Institute of Ecology and Evolution/Plant Biodiversity, Philosophenweg 16, 07743 Jena, Germany

**Keywords:** additive effect, common mullein, cross-resistance, environmental filter, greenhouse experiment, local adaptation, plant invasions, native vs. non-native populations, New Zealand, synergistic effect

## Abstract

During plant invasions, exotic species have to face new environmental challenges and are affected by interacting components of global change, which may include more stressful environmental conditions. We investigated an invasive species of New Zealand grasslands, commonly exposed to two concomitant and limiting abiotic factors—high levels of ultraviolet-B radiation and drought. The extent to which *Verbascum thapsus* may respond to these interacting stress factors via adaptive responses was assessed in a greenhouse experiment comprising native German plants and plants of exotic New Zealand origins. Plants from both origins were grown within four treatments resulting from the crossed combinations of two levels of UV-B and drought. Over twelve weeks, we recorded growth, morphological characteristics, physiological responses and productivity. The results showed that drought stress had the strongest effect on biomass, morphology and physiology. Significant effects of UV-B radiation were restricted to variables of leaf morphology and physiology. We found neither evidence for additive effects of UV-B and drought nor origin-dependent stress responses that would indicate local adaptation of native or exotic populations. We conclude that drought-resistant plant species might be predisposed to handle high UV-B levels, but emphasize the importance of setting comparable magnitudes in stress levels when testing experimentally for antagonistic interaction effects between two manipulated factors.

## 1. Introduction

Biological plant invasions are a key aspect of global change [1] and their mechanisms and preconditions have been frequently investigated to date [2,3,4]. A species has to overcome a number of barriers before it can be considered invasive elsewhere [5], among them biotic and abiotic conditions in the invaded range (see also [6]). Several mechanisms, including plastic and adaptive responses, which enable plant species to handle novel environmental conditions, have been repeatedly addressed. High phenotypic plasticity allows a genotype to develop different phenotypes in response to heterogeneous environments and is an often-observed advantageous property of invasive species, e.g., [7,8]. By contrast, pre-adaptation to particular environmental factors represented in single populations in the native range [9], as well as more recent adaptive evolution to novel environments following natural selection in the invaded range, can strongly contribute to a species’ invasive potential [10,11].

Addressing the role of large-scale abiotic factors as environmental barriers during plant invasions has so far mostly dealt with climatic conditions in native and invaded ranges and associated climatic niches of invasive species based on temperature and humidity tolerance [12,13]. Overall irradiation and biologically active UV-B radiation levels are equally subjected to climate change and are becoming more important for both resident plant communities and plant invasions [14]. However, these factors have been largely neglected in plant invasion research (see however: [15,16,17]). The effects of global change have largely been under consideration to date as ‘one-factor-only’ approaches. However, more recent research ambitions have identified the importance of testing for the role of interacting environmental factors [18,19,20,21].

The single effects of two environmental factors might be more or less linked so that their combined effect on plants cannot be directly extrapolated from plant response to each stress applied individually [22]. Two abiotic factors generally interact either in an additive, synergistic or antagonistic way [21,23]. A detrimental synergy of two limiting factors occurs, for instance, if the magnitude of the combined effect of both stressors exceeds the sum of the single stressor effects, as it has been observed, e.g., for jointly applied drought and heat stress on plants (see [22]). Another presumable scenario is an antagonistic interaction of stressors—a so-called cross-resistance—to both stresses by a decrease of sensitivity to one environmental factor during exposure to the other as it was previously described, e.g., in the field of biotic interactions of plants with herbivores and pathogens [24]. In natural habitats, some climatic factors are typically coupled, e.g., high solar radiation and high temperature [25]. The individual and combined contributions of these factors to plant responses can only be quantified experimentally in controlled environments. 

High radiation levels and high growing season temperatures are characteristic for temperate grasslands at the global scale and they often occur in combination with low water availability. Distinctively higher levels of UV-B radiation additionally apply to the Southern hemisphere when compared to comparable sites in the Northern hemisphere where many invasive plants originate [26]. High levels of UV-B affect several plant responses more rapidly and with stronger effects in herbaceous plant species than in woody species [27]. Therefore, in particular, grassland ecosystems can be supposed to show strong responses to UV-B and should receive more attention as UV-B radiation levels continue to vary and thus to be a component of global change [28].

UV-B radiation causes interferences at different organizational levels of plants, including DNA damage, limitation of photosynthesis and morphological changes due to decreasing phytohormone concentrations (e.g., IAA [29]). Consequently, UV-B-exposed plants suffer from reductions in biomass, height and leaf area [29,30,31] and experience changes in functional leaf traits, e.g., an increasing leaf dry matter content [16]. Effective UV-B protection can be provided by strengthening epidermal or cuticular structures and trichomes on the upper leaf surface [32,33], as well as by the incorporation of UV-B absorbing flavonoids and anthocyanins [29]. These protection measures can also be advantageous in regulating plants’ water balance [34]. In their review of effects of drought stress in plants, Jaleel et al. [35] identified drought as one of the most important abiotic environmental stress factors and described several effects on plants that are similar to consequences of UV-B, including biomass reduction, decreases in plant height and leaf area and changes in dry matter content and photosynthetic pigments.

The effects of drought and high UV-B intensities on plants have been frequently investigated individually, but have been addressed in combination less often. Additive detrimental effects of UV-B radiation and drought were shown, for example, for *Populus cathayana* [36] in terms of plant height and leaf area reduction, such as for total biomass decrease of the shrub *Hippophae rhamnoides* [37]. In a soybean (*Glycine max*) study, there was no evidence for additive effects of both abiotic factors in growth response and seed yield [38]. However, results of other studies indicated antagonistic effects of UV-B and drought in crops [39], in European heathland species [40] and even in conifer species [41,42]. Both environmental factors provoke an oxidative burst and, thus, can jointly induce protective measures. Moreover, several studies even revealed different adaptive responses of congeneric species or distinct populations within species dependent on local conditions of their origin. Comparing high altitude and low altitude *Populus* species (*P. kangdingensis*, *P. cathayana*) or *Hippophae rhamnoides* populations, respectively, high altitude individuals exhibited higher tolerance to drought in the presence of UV-B, whereas low altitude individuals showed additive damaging effects [36,37]. Consistent with these results, Hofmann et al. [43] found higher physiological acclimation capacity of stress-adapted slow growing *Trifolium repens* ecotypes under high UV-B radiation compared to other populations. UV-B × drought interactions have been predominantly examined for crops and woody species, but not yet considered with regard to plant invasions into grasslands.

**Figure 1:**
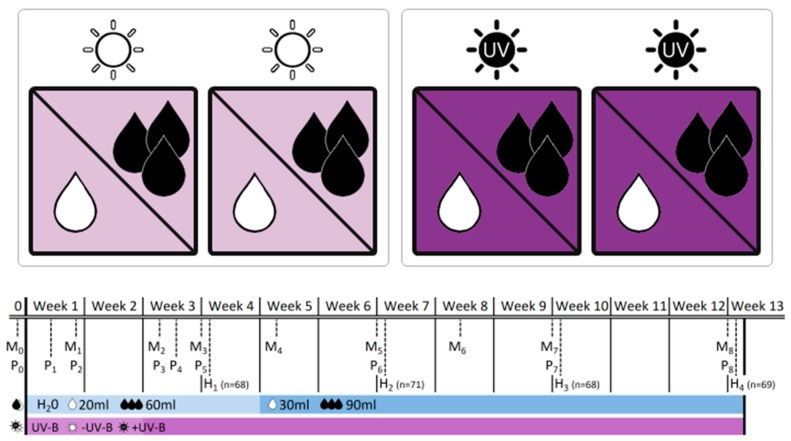
Experimental design of the greenhouse experiment indicating the four experimental boxes and the crossed application of ultraviolet-B (UV-B levels: 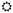 −UV-B/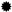 +UV-B) and water treatment (water levels: 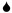 low/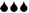 well-watered). The table provides information on the dates of monitoring (M_x_), physiological measurements (P_x_) and biomass harvests (H_x_) during the 12-week experimental period, and adds information on the treatment intensity applied. Note that levels of water addition were adjusted after 4 weeks to account for rapid initial increase in plant size.

In the present study, we compared native (German) and invasive (New Zealand) populations of the grassland species *Verbascum thapsus* L. in response to combined drought stress and UV-B radiation in a greenhouse experiment (Figure 1). We tested for pre-adaptation to UV-B radiation in native populations from Germany as being induced by high drought tolerance, and for recent local adaptation of exotic populations from New Zealand in growth and physiological responses. We addressed the hypotheses that i) the combined stress of UV-B and drought has an antagonistic effect on plants and ii) New Zealand populations are better adapted to high UV-B levels providing evidence of recent adaptation. To the best of our knowledge, this is the first study addressing the role of combined environmental stress of UV-B and drought for native and exotic origins in the context of plant invasion processes in the southern hemisphere.

## 2. Results

### 2.1. Biomass Data (Harvest Data H_x_)

**Figure 2:**
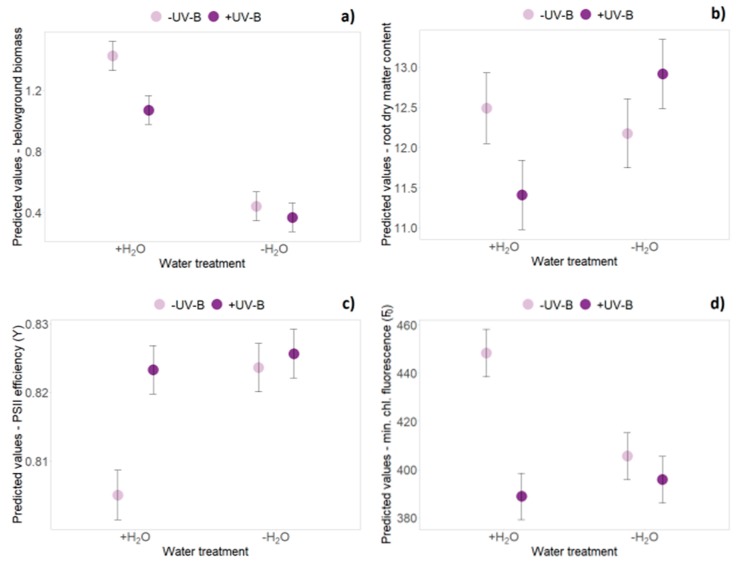
Interaction effects of UV-B and water treatment when averaging over origin. Predicted values ± SE of (**a**) belowground biomass, (**b**) root dry matter content, (**c**) PSII efficiency (Y) and (**d**) minimum chlorophyll fluorescence (F_0_) are shown across all treatment combinations (+H_2_O|-UV-B, +H_2_O|+UV-B, -H_2_O|-UV-B, -H_2_O|+UV-B).

At the first harvest, aboveground, belowground and total biomasses of German individuals were significantly higher (+26% on average), than biomass of exotic individuals (*p* < 0.05, Table 1 and Table S1). Overall, productivity was not significantly affected by UV-B radiation at any time point. Aboveground, belowground and total biomass were significantly reduced by about 40%–50% due to limited water availability at all four harvest dates (*p* < 0.001, Table 1 and Table S1). By contrast, water limitation significantly increased the shoot:mass ratio among all harvest dates, as well as root dry matter content at the first and the third harvest (Table 1). At the fourth harvest, German individuals showed a more pronounced decrease in belowground and total biomass, and a consequently stronger increase in the shoot:mass ratio under limited water availability conditions compared with New Zealand individuals, as evidenced by a significant origin × water treatment interaction (*p* < 0.05, Table 1 and Table S1). Significant interaction effects of UV-B treatment and water treatment occurred only at the second harvest date (Table 1): when averaging over native and exotic origin, an UV-B-induced reduction of belowground biomass was less apparent under additional water limitation (*p* = 0.012, Figure 2a, Table S1). In well-watered conditions, a decrease of root dry matter content (−9% on average) was observed under UV-B exposure, whereas an increase of root dry matter content (+6% on average) was caused by the combined application of UV-B and limited water availability (*p* = 0.032, Figure 2b, Table S1).

### 2.2. Growth Data (Monitoring Data)

**Figure 3:**
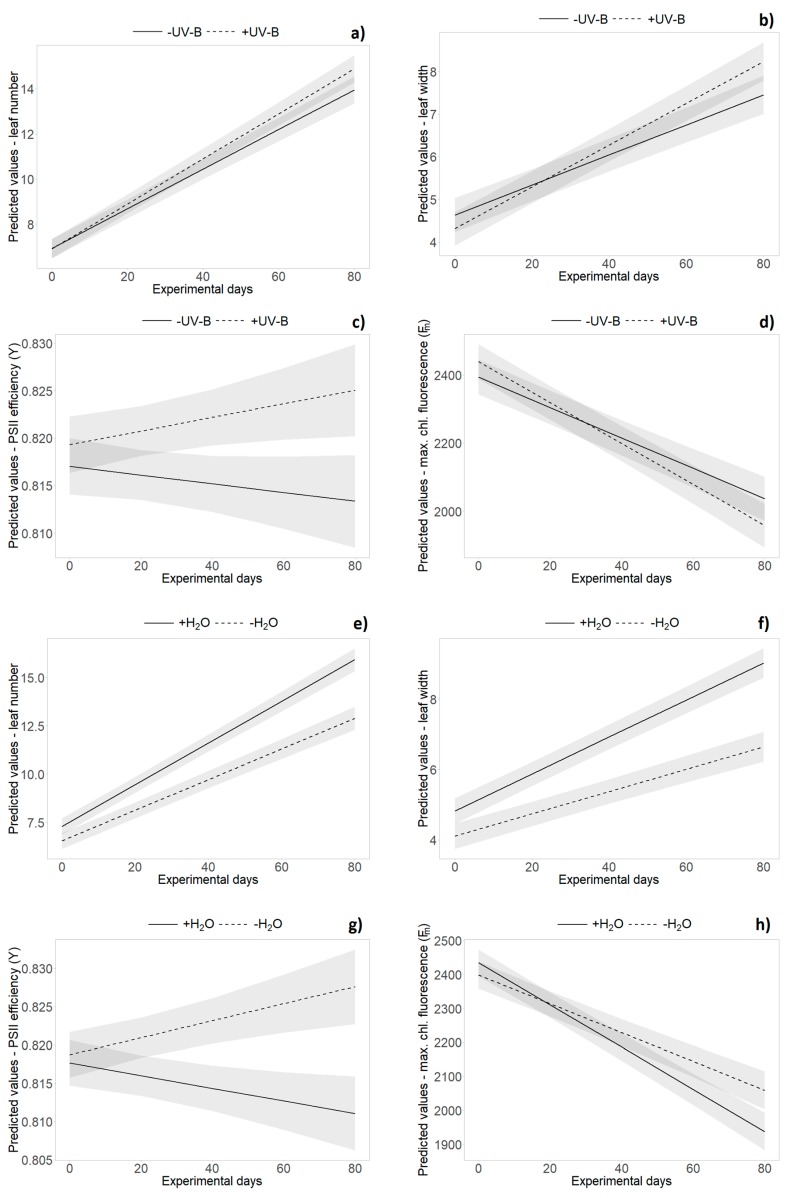
Effects of UV-B (**a–d**) and water treatment (**e–h**) over time. Predicted values ± SE of (**a,e**) leaf number, (**b,f**) leaf width, (**c,g**) PSII efficiency (Y) and (**d,h**) maximum chlorophyll fluorescence (F_m_) are shown in (**a–d**) absence of UV-B (solid line) and in presence of UV-B (dashed line), as well as under (**e–h**) well-watered conditions (solid line) and under drought (dashed line).

The repeated measures analysis across all monitoring dates during the experiment revealed an overall significantly higher leaf number, longer and wider leaves and bigger rosettes in German individuals compared to New Zealand plants (Table 2 and Table S2). Significant origin × time interactions revealed relatively faster leaf growth and rosette expansion of New Zealand individuals during the course of the experiment, whereas German plants were bigger in the beginning of the experiment (Table 2 and Table S2). The repeated measures analysis did not reveal significant main effects of “UV-B treatment” on any of the tested variables, but did reveal significant interaction effects with “time” for leaf number and leaf width. Leaf number and leaf width showed a stronger increase in the presence of UV-B radiation during the experiment (Table 2 and Table S2, Figure 3a,b). Single UV-B effects (e.g., reduced maximum leaf length (H_1_), rosette area (H_1_) and leaf number (H_2_)) or interaction effects with the origin (percentage of dead leaves (H_1_) and leaf number (H_3_)) were not consistent over time (see Tables S1 and S3).

Overall, leaf number and the proportions of dead leaves, leaf length and leaf width, as well as rosette size, were significantly decreased by the water limitation treatment (*p* < 0.001, Table 2 and Table S2, Figure 3e,f). Water level × origin interaction effects were only found at separate monitoring dates for various variables (e.g., leaf length (H_2_, H_4_), leaf width (H_3_) and leaf number (H_4_), see Table S3). The repeated measures analysis did not reveal significant interaction effects of “UV-B treatment” and “water treatment”, nor was there a three-way interaction with “origin” or “time” (Table 2).

### 2.3. Functional Leaf Traits and Physiology

Significant origin effects were found at separate harvest dates and as consistent effects evidenced over time: leaves of German individuals showed an about 10% higher leaf dry matter content (LDMC) than leaves of New Zealand individuals at the first two harvests (Table 1 and Table S1). Overall, PSII efficiency (Y) was significantly higher in German individuals (*p* = 0.004, Table 2 and Table S2), while minimum chlorophyll fluorescence (F_0_) was higher in New Zealand individuals (*p* = 0.005, Table 2 and Table S2). Maximum chlorophyll fluorescence (F_m_) decreased during the experiment with a stronger decline in German plants (*p* = 0.004, Table 2 and Table S2).

**Figure 4:**
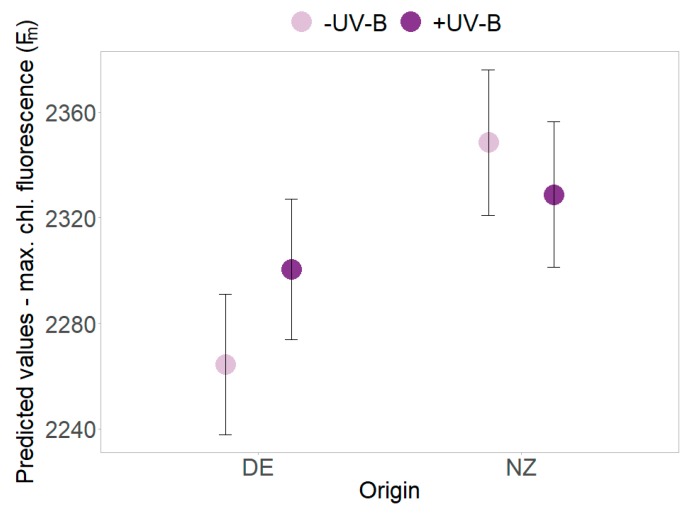
Interaction effects of UV-B and origin. Predicted values ± SE of maximum chlorophyll fluorescence (F_m_) are shown in the absence of UV-B (pale violet) and in the presence of UV-B (dark violet) for native German (DE) and exotic New Zealand individuals.

UV-B effects were found in PSII efficiency (Y) that decreased in the absence of UV-B but increased for plants under UV-B exposure (*p* = 0.018, Table 2 and Table S2, Figure 3c). The decrease of minimum and maximum chlorophyll fluorescence over time was more pronounced in presence of UV-B radiation (Table 2 and Table S2, Figure 3d). In presence of UV-B, overall higher maximum chlorophyll fluorescence (F_m_) of New Zealand individuals (slightly) decreased, whereas an increase was found for German plants (*p* = 0.011, Table 2 and Table S2, Figure 4). Further UV-B × origin interaction effects were found for single harvest dates (see Tables S1 and S3).

Water limitation significantly increased leaf dry matter content and PSII efficiency among all harvests. Overall, maximum chlorophyll fluorescence (F_m_) was increased under dry conditions, whereas higher values of minimum chlorophyll fluorescence (F_0_) were found for well-watered plants (*p* < 0.05, Table 2 and Table S2). PSII efficiency (Y) decreased with sufficient water availability but increased for plants under water limitation (*p* < 0.001, Table 2 and Table S2, Figure 3g). The decrease of maximum chlorophyll fluorescence (F_m_) over time was more pronounced in well-watered individuals (*p* < 0.001, Table 2 and Table S2, Figure 3h). For most of the variables, there was no significant interaction effect of origin and water, while insufficient water supply induced an increase of specific leaf area (H_4_) in New Zealand individuals only (see Table S3).

**Figure 5:**
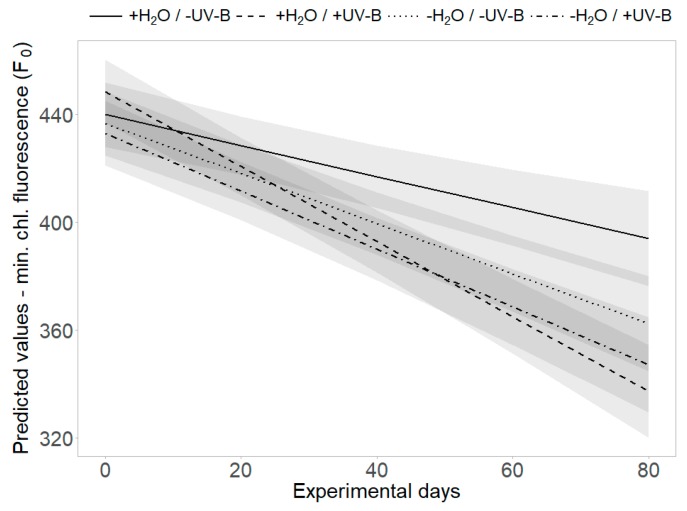
Interaction effects of UV-B and water treatment. Predicted values of minimum chlorophyll fluorescence (F_0_) are shown across all treatment combinations (+H_2_O|-UV-B, +H_2_O|+UV-B, -H_2_O|-UV-B, -H_2_O|+UV-B). Shaded areas depict the respective SE confidence intervals.

The threefold interaction effect of “UV-B treatment”, “water treatment” and “time” revealed a significant difference in minimum chlorophyll fluorescence (F_0_): the strongest decrease of F_0_ was found in the presence of UV-B radiation and the decrease was lessened by limited water availability, applied separately or in combination with UV-B radiation (*p* = 0.011, Table 2, Figure 5). At the second harvest, PSII efficiency (Y) was significantly increased and minimum chlorophyll fluorescence (F_0_) significantly decreased by UV-B radiation and low water availability individually, but this effect was not additionally enhanced by the joint presence of both factors (*p* < 0.01, Figure 2c,d).

## 3. Discussion

### 3.1. Single and Combined Effects of UV-B and Drought

The applied UV-B radiation treatment to both origins of *V. thapsus* aimed at simulating midsummer UV-B levels of the invaded range in New Zealand. In consequence, the applied UV-B intensity was familiar to the level exotic populations experience but novel to native individuals only. The observed limiting effects of UV-B radiation on leaf number, leaf length and rosette area confirm previous studies, which also reported UV-B-induced growth and biomass reduction [17,19,29,44]. A UV-B-induced increase in PSII efficiency was determined in the present study and caused by a decrease of minimum and maximum chlorophyll fluorescence (see also [45]). PSII efficiency response to UV-B has been previously identified for several species and was mostly found to decrease as a result of increasing minimum and maximum chlorophyll fluorescence [30,33,46] (but see also [47]). Especially an increase of minimum chlorophyll indicates photoinhibition and direct damage or an inactivation of PSII reaction centers as a result of a disconnection of light-harvesting antennae from their reaction centers [48,49,50]. A concomitant decrease of maximum and minimum chlorophyll fluorescence, as observed in the present study, was previously linked to thermal dissipation in PSII reaction centers, which displays a key photoprotective process [48]. In consequence, reactive oxygen species (ROS) production in response to a moderate dose of UV-B radiation in our experiment might be avoided and might even explain a temporary increase of PSII efficiency in the context of efficient repair mechanisms.

The applied water treatment presumably induced drought stress in the individuals of the “low” water treatment level, as those regularly responded with wilting of leaves at the latest on the day of watering. We therefore assume that a physiological stress response was provoked in the plants by water limitation. An overall reduced biomass, leaf number, leaf area and higher leaf dry matter content confirm this assumption and have been previously observed in response to water limitation (e.g., [35,44,51]). In line with the effects of UV-B, the maximum photosynthetic quantum yield of all plants was higher under drought conditions. This effect was initially caused by a decrease of minimum chlorophyll fluorescence, and in the later experimental phase due to an increase of maximum chlorophyll fluorescence. Previous studies on drought effects predominantly revealed decreasing PSII efficiency due to water limitation [52,53]. The opposite effect in the present study might be the consequence of the existing drought tolerance and resulting protection measures of *Verbascum thapsus*, that are known to naturally occur on very dry and disturbed sites [54].

We found no evidence for detrimental synergy effects or generally additive effects of drought and UV-B radiation as they had been reported before [36,37]. Other studies found enhanced drought tolerance in the presence of UV-B radiation, since certain stress avoidance mechanisms turned out to be of advantage under both abiotic stresses, e.g., leaf area reduction, increase of leaf cuticle thickness or stomatal closure [38,40,55]. Relevant antagonistic effects can be also provoked at the physiological level by common metabolic responses to drought and UV-B, e.g., an increase of anthocyanins, phenolics, prolin and other antioxidants to decrease ROS production and consequently maintain photosynthetic capacity and carbon assimilation rate [39,41,42,43].

In the present study, the only significant interaction effects of UV-B and drought were found after six weeks of the experiment (H_2_) and might have been the temporary consequence of the UV-B radiation dose increase after the initial two-week UV-B acclimation phase. Belowground biomass moderately decreased in response to UV-B radiation [16,56] but was highly sensitive to drought with a strong overall decrease [44] that was not additionally aggravated by supplementary UV-B exposure. By contrast, root dry matter content did not change significantly with drought in the absence of UV-B, but increased under combined stress application and decreased under UV-B exposure in well-watered conditions, thus displaying strong interaction effects. Dry matter distribution towards the roots has been previously shown for plant species under abiotic stress conditions [57,58], and might be only induced in response to the combined application of drought and UV-B in our study. Furthermore, the PSII efficiency was increased by UV-B and drought to a similar extent, whether applied separately or jointly. This points to a similar and non-additive effect size of both abiotic stresses at the physiological level. Interestingly, the minimum chlorophyll fluorescence (F_0_) appeared to be more sensitive in response to UV-B radiation at the second harvest (H_2_) and in the repeated measures analysis. The only explanation for a decrease of F_0_ might be a higher number of unimpaired PSII reaction centers that could be provided by activation of efficient photoprotection and repair mechanisms. Those might be induced to a higher level by UV-B compared to drought, as radiation displays the more immediate trigger for PSII damage. Therefore, we could conclude that the plant physiology of *V. thapsus* is affected by both UV-B and drought to a similar extent, but the respective effect is induced by different underlying mechanisms.

### 3.2. Origin Differentiation and Origin-Specific Response to UV-B and Drought

Differences in plant performance or functional plant traits between native and exotic origins might hint at genetic differentiation as a result of founder effects or evolutionary processes during the invasion of novel habitats in New Zealand [59]. As the set of investigated German and New Zealand populations does not represent the entire native and invaded range, respectively, other sources of variation among populations may have also contributed to significant differences between origins: among them are population-specific differences in elevation, microclimate or other environmental factors that are able to induce geographical clines within ranges [54,60]. Thus, complementary experiments with further seed material from other parts of the native and invaded range along larger latitudinal gradients would be necessary to draw more general conclusions. 

In the present study, native individuals from Germany started with higher biomass and larger leaves, but exotic individuals showed stronger increase in time regarding leaf area and respective size of rosettes. Therefore, the initial advantage of native plants disappeared during the experimental runtime. Higher productivity/relative growth rates of exotic populations in comparison to native origins has been repeatedly reported in the past, especially in the context of altered resource allocation as a result of the release from native biotic and abiotic stresses [2,54,61]. Nevertheless, in our study, the New Zealand populations of *V. thapsus* appeared to be less successful in the early establishment (personal observation). The difference in seed age of the German and New Zealand populations and the conditions during seed transfer from New Zealand to Germany might be a reason for differences in germination and establishment success. Further explanations for the initial disadvantage of exotic populations may comprise a potentially reduced genetic diversity of exotic populations due to founder effects or the importance of range identity with regard to covarying effects of different biotic and abiotic conditions, as discussed by Dieskau et al. [62]. This might be particularly important, since other native–invasive comparisons revealed early invasive superiority when testing *V. thapsus* performance in other parts of the species’ exotic range [54,63,64]. PSII efficiency was found to be generally higher in native plants with a stronger decrease of maximum chlorophyll fluorescence (F_m_) in German individuals during the experiment and higher values of minimum chlorophyll fluorescence (F_0_) in New Zealand plants since the second harvest (H_2_) independent of water availability and UV-B treatment. Higher physiological performance of native individuals could also be a consequence of their early establishment success prior to the application of environmental stress by drought and UV-B radiation, which might have led to stronger and more resilient plants. 

In the presence of UV-B, New Zealand plants showed the described reduction of maximum chlorophyll fluorescence, whereas an observed increase of maximum chlorophyll fluorescence in German individuals might be linked to an impaired electron transfer or secondary electron acceptor of PSII [49]. In contrast to German plants, New Zealand individuals also showed a pronounced decrease of minimum chlorophyll fluorescence and an increase of leaf number and dead leaf proportion in the early stage of the experiment. Therefore, the higher photoprotection abilities and growth of exotic individuals under UV-B radiation might indicate an evolved reduced sensitivity to UV-B in consequence to the experienced higher radiation levels in New Zealand.

Drought stress is known to limit invasibility of habitats, as drier sites appear to be less invaded and non-natives turned out to be more abundant in wetter years [65]. Nevertheless, previous studies did not agree on the question if drought tolerance of native and non-native species differs and thus is subject to evolutionary changes in plant invasions [66,67,68]. In the present study, non-native plants from New Zealand responded with measurable changes in leaf morphology to low water availability, whereas native German plants experienced a stronger decrease in growth estimates in the late experimental phase. We could therefore assume that non-native genotypes are able to respond with functional changes at the leaf level in order to sustain overall growth under drought conditions. This ability might be the result of evolutionary processes in response to environmental conditions in the invaded range or overall higher phenotypic plasticity [69]. Interestingly, previous studies on drought tolerance of native and non-native populations of grassland species assumed a trade-off between rapid growth and drought tolerance, since they revealed more resilient native populations under drought conditions, although non-native populations appeared to be more vigorous and fast-growing in other environments [61,70]. 

Furthermore, we found no evidence for the importance of population origin to the combined stress effects on plants. Previous studies on different woody plant species revealed different resistance of low and high altitude populations to a treatment combining UV-B radiation and drought [36,37]: whereas low altitude populations experienced additive detrimental effects of both abiotic stressors on productivity and growth traits, high altitude populations responded with higher tolerance to the combined application of drought and UV-B, and thus appeared to be better adapted. By contrast, testing for adaptation to elevational constraints in multiple exotic plant species gradient, Watermann et al. [31] did not find any evidence for combined UV-B × drought interactions with low and high altitude populations. However, neither native nor non-native populations of *Verbascum thapsus* had an advantage in the presence of combined abiotic stress by drought and UV-B radiation in the present experiment. As both origins are expected to be similarly adapted to drought but experience different levels of UV-B in their home ranges, we assume that origin differences in stress response may be more precisely carved out by moderate water limitation and moderate or elevated UV-B intensity. While severe levels of stress usually lead to direct negative effects on plant metabolism and growth, moderate abiotic stress triggers physiological and biochemical defense mechanisms, which are of advantage under harmful conditions [44].

## 4. Materials and Methods

### 4.1. Study Species

*Verbascum thapsus* L. (Scrophulariaceae) is a typical component of temperate dry grasslands and ruderal habitats in the investigated ranges in Germany and New Zealand, and is characterized by high drought tolerance and a strong prevalence in open, unshaded habitats [71]. The species is monocarpic, generally biennial and develops a long tap root to access remote nutrient and water resources in deeper soil layers [72]. The plant’s surface is typically piliferous, i.e., leafs and stems are densely covered with woolly, branched stellate trichomes, which provide a reliable protection against herbivory, frost and drought [73] and may be also advantageous under high radiation levels. The native distribution of *V. thapsus* ranges from Europe to Central Asia. To date, it is furthermore naturalized in North America, Hawaii, Australia and New Zealand. In the present study, we used ten native and eight invasive populations of *V. thapsus* from Germany and New Zealand, respectively (for population details see Table S4).

### 4.2. Experimental Design

The experiment was conducted in the summer of 2013 in the greenhouse cabinets of the Martin Luther University Halle–Wittenberg. Seeds were germinated in the greenhouse under standard conditions within seedling trays on a soil-sand mixture (2:1) and transferred into pots (9 × 9 × 10 cm) with the same substrate about six weeks later. At the age of ten weeks, plants were assigned to the experimental setting: four treatments resulting from fully crossed combinations of two water levels (“low” vs. “well-watered”) and two UV-B levels (“−UV-B” vs. “+UV-B”) were applied to four individuals of all 18 populations (totaling 288 individuals). Therefore, plants were arranged within four identical boxes (120 × 120 × 70 cm), which served as self-contained UV-B environments (Figure 1). All boxes were equipped with white chipboard to the left and the right side and with white fleece at the front and the back, allowing the implementation of a UV-B radiation source from the top to the plants and to ensure ventilation within the boxes to minimize uncontrolled microclimatic effects. Each of the four boxes was equipped with a greenhouse PAR lamp (HQI 400 W, Philips) on the top. Additionally, in two boxes, three UV-B tubes (TL 20W/12 RS SLV, Philips) were implemented. The two boxes without UV-B tubes served as a “no UV-B” control. Photosynthetically active radiation (PAR) was applied 16 h a day, whereas UV-B lamps were switched on for eight hours within this period. Initially, UV-B lamps had a distance of 80 cm to the plant individuals, resulting in a UV-B intensity of 0.014-0.052 mW cm^−2^ dependent on pot position. After two weeks we reduced the distance between the lamps and the plants in order to increase UV-B radiation to 0.096–0.159 mW cm^−2^, thereby approaching the midsummer UV-B level on the South Island, New Zealand [17].

Half of the plants in each box received sufficient water supply, whereas the other half was exposed to drought (Figure 1). Both treatment groups were watered every second day with water amounts in a ratio of 3:1 (week 1–4: 60 mL/20 mL, week 5–12: 90 mL/30 mL, for well-watered and drought treatments, respectively). Measurements of soil moisture using a time-domain reflectometer (TDR) revealed water contents of 15%–20% in well-watered pots and 3%–8% in pots mimicking situations of drought. Based on a visual assessment, the latter group generally reached the wilting point within 48 h but was kept from being permanently damaged.

Individuals of each population were equally represented at each UV-B level and water level. Initially, all individuals were randomly assigned to and positioned within the boxes, but within-box randomization was subsequently repeated every 7–10 days during the experiment. Due to the occurrence of some mortality in the early phase of the study, we received data of 276 individuals of 18 populations (10 DE, 8 NZ) within an experimental period of 12 weeks.

### 4.3. Data Collection

Biometrical variables were determined for each individual on a weekly basis in the beginning, and later every ten days (Figure 1): rosette diameter, length and width of the longest leaf and the number of intact and dead leaves were recorded ten times during the experiment. Rosette area (A) was calculated for each individual as an ellipse using measured rosette diameters (d_1_, d_2_):

A = π × (d_1_/2) × (d_2_/2), (1)

In order to assess repeated productivity data and growth rates, one individual per population and treatment was harvested every three weeks, resulting in four harvests during the experimental period (*n* = 68–71, Figure 1). We determined aboveground, belowground and dead biomass, leaf dry matter content (LDMC), root dry matter content (RDMC), specific leaf area (SLA) and the shoot:root ratio for each individual harvested in the different subsets. The selective sampling for harvest reduced the total amount of individuals available for monitoring of biometrical variables over time. 

At the physiological level, we recorded maximum quantum yield of photochemical energy conversion (Y) as a measure of photosystem II efficiency, such as minimum and maximum fluorescence yield (F_0_, F_m_) in response to the initial UV-B application and the enhancement of UV-B intensity after a two-week acclimatization, totaling eight times during the experiment (Figure 1). Therefore, one fully developed and healthy leaf per individual was dark-adapted for about ten minutes and subsequently measured once with a hand-held fluorometer (Mini-PAM, Heinz Walz GmbH) without removal from the experimental boxes or interruption of the UV-B treatment.

### 4.4. Statistical Analysis

A repeated measures analysis of the monitoring data was done to test for the effects of origin, UV-B treatment and water treatment on plant performance over time in R (Version 3.5.3, R CORE TEAM 2019). We therefore applied a linear mixed effect model (function “lmer”, package “lmerTest”, [74]) containing “origin” (DE vs. NZ), “UV-B” (-UV-B vs. +UV-B) and “water” (low vs. well-watered) as fixed factors and “time” (eight dates of monitoring and physiological measurements), as well as all of their interactions. The following nested random effect terms were additionally included in the repeated measures analysis: “box:UV-B” and “population:origin”, while fitting a random slope model with “time|plant ID:UV-B:water:origin”. Due to the partial harvests during the experiment, the repeated measures analysis of the monitoring data naturally experienced a decrease in sample size over time. Thereby, the number of replicates within populations was reduced from four to one during the experimental duration. As the statistical analyses aim to test for differences between origins (DE vs. NZ), all remaining individuals of the ten German populations served as replicates for the origin level “DE” and all remaining individuals of the eight New Zealand populations were considered replicates for the origin level “NZ”. 

Furthermore, data of the four partial biomass harvests was separately analyzed per date by linear mixed effect models containing “origin” (DE vs. NZ), “UV-B” (-UV-B vs. +UV-B) and “water” (low vs. well-watered), as well as their interactions as fixed factors. We additionally included the individual leaf number at the time of the experimental start as a covariate and again determined “box:UV-B” and “population:origin” as nested random effect terms in the separate mixed effects models. 

## 5. Conclusions

Generally, interaction effects of UV-B and drought depend on species-specific sensitivity, stress factor intensity, exposure duration and operation mode [44]. In our study, the strong effect of water treatment might potentially mask UV-B effects on plants, as the induced water limitation level is likely to display a more restrictive condition for plant metabolism than the applied UV-B radiation level. This observation points at the importance of setting comparable stress levels in abiotic interaction experiments, as otherwise one of the abiotic environmental factors dominates the results and potential antagonistic effects are difficult to detect. Moreover, application of artificial UV-B under greenhouse conditions partly excludes photosynthetically active radiation (PAR) that is known to have a mitigating effect on plants under UV-B and might have additionally induced mechanisms of drought resistance [44,75]. Nevertheless, our results point at similar physiological responses to drought and UV-B radiation and an absence of detrimental synergy effects of both environmental factors. Therefore, we assume that drought-tolerant plant species might also be more resilient to higher levels of UV-B radiation. To adequately test and identify cross-resistance mechanisms in plant invasions and the potential impact of local adaptation on this characteristic, we recommend attaching great importance to the application of suitable and relevant environmental stress gradients derived from respective native and/or invaded ranges in future experimental studies.

## Figures and Tables

**Table 1:** Fixed-effect results of the harvest data analysis. “UV-B” and “Water” depict the effect of treatments and “Origin” refers to the effect of German vs. New Zealand provenance. Degrees of freedom (df_N_ = numerator, df_D_ = denominator), F statistics (F) and significance values (p) are provided. Significant p-values (* *p* < 0.05; ** *p* < 0.01; *** *p* < 0.001) and marginal effects (. *p* < 0.1) are indicated.

Variable/Source	df_N_	1st Harvest (3 Weeks)				2nd Harvest (6 Weeks)				3rd Harvest (9 Weeks)				4th Harvest (12 Weeks)			
		df_D_	F	*p*		df_D_	F	*p*		df_D_	F	*p*		df_D_	F	*p*	
***Total biomass***																	
Origin	1	57.1	5.449	0.023	*	17.9	2.415	0.138		23.2	0.146	0.706		18.9	2.929	0.103	
UV-B	1	1.3	0.001	0.984		2.0	0.483	0.560		44.5	2.448	0.125		45.2	0.064	0.801	
Water	1	58.3	30.522	<0.001	***	43.6	156.263	<0.001	***	44.7	85.480	<0.001	***	45.3	123.432	<0.001	***
Initial leaf number (Covariate)	1	57.2	30.682	<0.001	***	34.6	18.697	<0.001	***	58.6	14.589	<0.001	***	59.4	16.040	<0.001	***
Origin × UV-B	1	57.0	0.185	0.669		44.2	0.047	0.829		44.6	0.250	0.619		45.1	0.318	0.575	
Origin × Water	1	26.6	0.797	0.380		44.0	3.692	0.061	.	44.6	0.164	0.688		45.2	4.241	0.045	*
UV-B × Water	1	58.3	0.001	0.975		44.5	0.809	0.373		44.7	0.762	0.387		45.1	0.617	0.436	
Origin × UV-B × Water	1	26.2	0.238	0.629		43.3	0.098	0.755		44.6	0.020	0.888		46.7	0.195	0.661	
***Aboveground biomass***																	
Origin	1	57.1	5.281	0.025	*	18.5	1.777	0.199		23.2	0.064	0.802		18.5	1.430	0.247	
UV-B	1	1.3	0.036	0.875		43.6	0.118	0.733		44.7	0.628	0.432		44.8	0.075	0.786	
Water	1	58.3	26.053	<0.001	***	45.3	82.856	<0.001	***	44.8	58.462	<0.001	***	44.9	49.639	<0.001	***
Initial leaf number (Covariate)	1	57.2	28.713	<0.001	***	37.8	12.078	0.001	**	58.2	16.458	<0.001	***	59.3	7.827	0.007	**
Origin × UV-B	1	57.0	0.504	0.480		46.2	0.000	0.988		44.7	0.316	0.577		44.7	0.441	0.510	
Origin × Water	1	25.4	0.297	0.590		46.3	0.926	0.341		44.7	0.078	0.782		44.8	0.088	0.768	
UV-B × Water	1	58.3	0.004	0.952		46.3	0.000	0.988		44.8	0.175	0.678		44.8	1.862	0.179	
Origin × UV-B × Water	1	25.1	0.464	0.502		45.7	0.011	0.918		44.7	0.055	0.816		46.1	0.051	0.823	
***Belowground biomass***																	
Origin	1	57.1	4.137	0.047	*	17.8	2.915	0.105		23.9	0.100	0.755		18.9	4.035	0.059	.
UV-B	1	1.2	0.471	0.602		2.0	2.905	0.232		1.7	1.413	0.374		1.7	0.353	0.620	
Water	1	58.6	33.416	<0.001	***	44.3	250.101	<0.001	***	44.8	83.539	<0.001	***	45.9	135.924	<0.001	***
Initial leaf number (Covariate)	1	57.3	26.224	<0.001	***	28.5	29.625	<0.001	***	57.0	9.803	0.003	**	51.3	16.662	<0.001	***
Origin × UV-B	1	57.0	0.160	0.691		45.0	0.574	0.453		42.8	0.109	0.742		43.8	0.126	0.724	
Origin × Water	1	16.5	3.488	0.080	.	45.0	12.119	0.001	**	44.8	2.071	0.157		45.7	11.764	0.001	**
UV-B × Water	1	58.6	0.084	0.773		45.0	6.936	0.012	*	44.9	0.347	0.559		45.7	0.015	0.903	
Origin × UV-B × Water	1	16.2	0.091	0.766		44.3	0.462	0.500		44.9	0.008	0.928		47.6	0.649	0.424	
***Shoot:mass ratio***																	
Origin	1	17.1	0.195	0.664		20.0	0.946	0.342		57.4	0.837	0.364		16.2	0.870	0.365	
UV-B	1	44.9	3.788	0.058	.	1.9	8.108	0.108		1.8	1.405	0.368		1.7	6.740	0.141	
Water	1	45.9	4.413	0.041	*	45.8	69.580	<0.001	***	59.0	95.446	<0.001	***	42.8	463.820	<0.001	***
Initial leaf number (Covariate)	1	47.2	1.465	0.232		38.8	0.421	0.520		57.9	0.0225	0.637		59.2	14.310	<0.001	***
Origin × UV-B	1	44.8	0.765	0.386		46.2	0.007	0.933		57.0	0.217	0.643		39.6	1.700	0.200	
Origin × Water	1	44.9	2.113	0.153		46.0	0.028	0.868		59.0	0.319	0.575		42.3	4.660	0.037	*
UV-B × Water	1	45.5	0.005	0.944		46.7	0.714	0.402		59.0	0.045	0.833		42.6	0.040	0.845	
Origin × UV-B × Water	1	44.7	0.437	0.512		45.3	0.086	0.771		59.0	0.281	0.598		44.2	3.670	0.062	.
***Root dry matter content***																	
Origin	1	57.1	0.085	0.772		61.2	4.562	0.037	*	59.0	0.912	0.344		58.1	0.011	0.919	
UV-B	1	1.2	0.856	0.503		1.9	0.057	0.835		59.0	0.964	0.330		59.7	3.427	0.069	.
Water	1	58.5	17.623	<0.001	***	61.7	1.998	0.162		59.0	4.820	0.032	*	59.7	0.791	0.377	
Initial leaf number (Covariate)	1	57.3	5.020	0.029	*	61.9	0.002	0.961		59.0	0.602	0.441		59.7	5.606	0.021	*
Origin × UV-B	1	57.0	0.893	0.349		60.4	1.354	0.249		59.0	0.234	0.630		59.7	0.562	0.456	
Origin × Water	1	17.6	0.002	0.966		60.0	0.037	0.848		59.0	3.678	0.060	.	59.7	0.823	0.368	
UV-B × Water	1	58.5	0.001	0.974		61.7	4.828	0.032	*	59.0	0.610	0.438		59.7	0.832	0.365	
Origin × UV-B × Water	1	17.2	0.929	0.349		60.0	0.002	0.961		59.0	0.004	0.984		59.8	1.349	0.250	
***Leaf dry matter content***																	
Origin	1	56.1	5.683	0.021	*	62.0	14.119	<0.001	***	21.3	0.044	0.836		16.9	0.623	0.441	
UV-B	1	1.0	4.180	0.294		62.0	0.977	0.327		1.2	0.000	0.994		1.7	0.004	0.957	
Water	1	57.4	56.591	<0.001	***	62.0	19.869	<0.001	***	31.4	16.113	<0.001	***	44.0	8.474	0.006	**
Initial leaf number (Covariate)	1	55.9	44.973	<0.001	***	62.0	5.653	0.021	*	54.2	4.123	0.047	*	52.7	4.903	0.031	*
Origin × UV-B	1	56.1	0.577	0.451		62.0	0.030	0.863		41.8	0.173	0.680		42.8	0.050	0.824	
Origin × Water	1	10.3	0.102	0.756		62.0	0.405	0.527		31.8	0.321	0.576		43.8	2.840	0.099	.
UV-B × Water	1	57.4	0.907	0.345		62.0	2.773	0.101		30.2	0.452	0.506		43.8	0.656	0.422	
Origin × UV-B × Water	1	10.1	1.161	0.306		62.0	0.173	0.679		30.0	0.818	0.373		46.0	0.037	0.849	
***Specific leaf area***																	
Origin	1	59.0	0.402	0.528		20.4	0.080	0.780		20.8	3.013	0.097	.	18.1	1.193	0.289	
UV-B	1	59.0	0.110	0.741		46.6	1.327	0.255		1.8	0.560	0.540		44.4	0.005	0.944	
Water	1	59.0	2.638	0.110		46.6	4.553	0.038	*	42.5	6.955	0.012	*	44.5	3.259	0.078	.
Initial leaf number (Covariate)	1	59.0	19.424	<0.001	***	42.1	0.762	0.388		52.9	5.081	0.028	*	59.3	0.157	0.694	
Origin × UV-B	1	59.0	0.062	0.805		47.5	0.003	0.957		41.0	1.401	0.243		44.3	0.993	0.324	
Origin × Water	1	59.0	0.165	0.686		47.6	0.552	0.461		42.6	0.405	0.528		44.4	10.525	0.002	**
UV-B × Water	1	59.0	0.569	0.454		47.6	0.099	0.755		42.8	1.647	0.206		44.4	2.822	0.100	.
Origin × UV-B × Water	1	59.0	1.626	0.207		47.0	0.059	0.810		42.9	0.520	0.475		46.1	1.662	0.204	

**Table 2:** Fixed-effect results of the repeated measures analysis. “UV-B” and “Water” depict the effect of treatments and “Origin” refers to the effect of German vs. New Zealand provenance. Degrees of freedom (df_N_ = numerator, df_D_ = denominator), F statistics (F) and significance values (p) are provided. Significant p-values (* *p* < 0.05; ** *p* < 0.01; *** *p* < 0.001) and marginal effects (. *p* < 0.1) are indicated.

**Source**	**df_N_**	**Leaf Number**				**Leaf Length**				**Leaf Width**				**Proportion of Dead Leaves**			
		**df_D_**	**F**	**p**		**df_D_**	**F**	**p**		**df_D_**	**F**	**p**		**df_D_**	**F**	**p**	
Origin	1	15.61	10.01	0.0062	**	34.3	56.710	<0.001	***	18.601	20.91	<0.001	***	15.9	3.120	0.096	.
UV-B	1	2.911	0.05	0.8427		4.9	2.240	0.196		3.059	2.33	0.2228		2.7	0.990	0.401	
Water	1	236.17	26.89	<0.001	***	231.8	46.890	<0.001	***	248.68	41.32	<0.001	***	252.9	11.510	<0.001	***
Time	1	200.54	1524.66	<0.001	***	115.5	558.000	<0.001	***	147.23	686.36	<0.001	***	169.2	2727.47	<0.001	***
Origin × UV-B	1	236.96	0.19	0.6646		232.4	0.160	0.693		249.35	1.77	0.185		253.6	0.360	0.547	
Origin × Water	1	236.14	0.27	0.6069		231.8	0.410	0.520		248.55	0.06	0.812		252.9	1.230	0.2684	
UV-B × Water	1	236.7	0.06	0.8051		232.2	0.750	0.389		249.18	0.82	0.3659		253.3	0.770	0.3811	
Origin × Time	1	200.54	0.2	0.657		115.5	38.860	<0.001	***	147.24	13.99	<0.001	***	169.2	0.040	0.8368	
UV-B × Time	1	200.55	6.4	0.0122	*	115.5	0.070	0.7947		147.18	15.7	<0.001	***	169.2	0.320	0.570	
Water × Time	1	200.55	33.43	<0.001	***	115.6	36.070	<0.001	***	147.3	42.43	<0.001	***	169.2	20.530	<0.001	***
Origin × UV-B × Water	1	236.66	2.77	0.0975	.	232.2	0.530	0.467		249.05	0.09	0.7702		253.4	0.310	0.577	
Origin × UV-B × Time	1	200.55	0.04	0.8459		115.5	0.510	0.478		147.2	2.16	0.1435		169.2	2.930	0.089	.
Origin × Water × Time	1	200.54	1.3	0.2554		116.1	1.700	0.195		147.96	2.03	0.1564		169.2	0.150	0.703	
UV-B × Water × Time	1	200.55	0.05	0.8263		115.6	0.050	0.823		147.29	2.97	0.0871	.	169.2	0.070	0.785	
Origin × UV-B × Water × Time	1	200.54	0.01	0.9082		116.1	0.870	0.353		147.95	0.17	0.6786		169.2	0.050	0.817	
**Source**	**df_N_**	**Rosette Area**				**PSII Efficiency (Y)**				**Min. Chlorophyll Fluorescence**				**Max. Chlorophyll Fluorescence**			
		**df_D_**	**F**	**p**		**df_D_**	**F**	**p**		**df_D_**	**F**	**p**		**df_D_**	**F**	**p**	
Origin	1	20.841	26.55	<0.001	***	21.6	10.259	0.004	**	20.5	10.079	0.005	**	27.5	1.770	0.195	
UV-B	1	3.879	1.51	0.2889		217.0	2.368	0.125		3.0	0.115	0.756		2.3	1.240	0.367	
Water	1	255	43.67	<0.001	***	215.9	0.461	0.498		226.5	5.354	0.022	*	234.6	5.690	0.018	*
Time	1	202.01	1351.2	<0.001	***	138.7	0.670	0.414		148.1	257.314	<0.001	***	170.9	420.720	<0.001	***
Origin × UV-B	1	255.72	0.08	0.7768		217.2	1.239	0.267		228.1	0.52	0.472		235.9	6.490	0.011	*
Origin × Water	1	254.9	0.35	0.5528		215.9	0.472	0.493		226.3	2.293	0.131		234.2	2.330	0.128	
UV-B × Water	1	255.53	2.79	0.0961	.	216.5	0.015	0.903		227.2	2.295	0.131		235.2	3.040	0.082	.
Origin × Time	1	202.02	21.99	<0.001	***	138.7	2.588	0.110		148.1	2.839	0.361		170.9	8.300	0.004	**
UV-B × Time	1	202.01	0.18	0.6752		138.6	5.691	0.018	*	148.0	15.478	<0.001	***	170.8	9.030	0.003	**
Water × Time	1	202.06	176.111	<0.001	***	138.6	16.625	<0.001	***	148.0	0.222	0.638		170.8	14.080	<0.001	***
Origin × UV-B × Water	1	255.44	0.44	0.5059		216.5	2.947	0.087	.	227.0	0.823	0.365		234.8	0.820	0.367	
Origin × UV-B × Time	1	202.01	0.69	0.4085		138.6	0.067	0.797		148.0	0.004	0.949		170.8	0.400	0.530	
Origin × Water × Time	1	202.3	3.82	0.0519	.	138.6	1.301	0.256		148.0	3.853	0.052	.	170.7	1.450	0.230	
UV-B × Water × Time	1	202.06	0.12	0.733		138.6	1.778	0.185		148.0	6.593	0.011	*	170.8	2.120	0.147	
Origin × UV-B × Water × Time	1	202.3	1.01	0.3152		138.6	0.434	0.511		148.0	1.119	0.292		170.7	0.170	0.679	

## Supplementary Materials

The following are available online at https://www.mdpi.com/2223-7747/9/2/269/s1, Table S1: Effect directions of harvest data analysis; Table S2: Effect directions of repeated measures analysis; Table S3: Fixed-effect results of the harvest data analysis; Table S4: Location and sampling information of native (German) and exotic (New Zealand) populations included in the experiment.
