# Supplementary material for: Combined Effects of UV-B and Drought on Native and Exotic Populations of Verbascum thapsus L
Source: Plants (Basel). 2020 Feb 18;9(2):269. doi: 10.3390/plants9020269 (PMC7076424; doi:10.3390/plants9020269)
Supplement: Supplementary file 1 [file plants-09-00269-s001.pdf]

Supplementary Materials

Table S1: Effect directions of harvest data analysis. Predicted values (‘fit’), standard error (‘SE’) and confidence interval (‘lower’ and ‘upper’ limits) are given for all significant main and twofold interaction effects of ‘Origin’, ‘UV-B’, and ‘Water’ in the harvest data analysis. Significance levels of effects are indicated by asterisks (*P < 0.05; **P < 0.01; ***P < 0.001).

		1st harvest (3 weeks)					2nd harvest (6 weeks)					3rd harvest (9 weeks)					4th harvest (12 weeks)				
Total biomass																					
Origin		fit	se	lower	upper	*															
DE		1.523	0.098	1.327	1.720																
NZ		1.205	0.114	0.977	1.432																
Water		fit	se	lower	upper	***	fit	se	lower	upper	***	fit	se	lower	upper	***	fit	se	lower	upper	***
well		1.756	1.102	1.552	1.961		3.716	0.156	3.404	4.027		5.667	0.252	5.163	6.171		8.010	0.364	7.281	8.739	
low		1.028	1.102	0.824	1.233		1.758	0.155	1.449	2.067		2.892	0.259	2.375	3.409		3.462	0.368	2.725	4.199	
Origin x Water																	fit	se	lower	upper	*
DE	well																8.858	0.500	7.858	9.858	
NZ	well																6.907	0.561	5.784	8.030	
DE	low																3.601	0.497	2.607	4.595	
NZ	low																3.281	0.578	2.125	4.436	
Aboveground biomass																					
Origin		fit	se	lower	upper	*															
DE		1.155	0.074	1.007	1.303																
NZ		0.915	0.086	0.743	1.088																
Water		fit	se	lower	upper	***	fit	se	lower	upper	***	fit	se	lower	upper	***	fit	se	lower	upper	***
well		1.311	0.077	1.156	1.465		2.468	0.100	2.268	2.668		3.746	0.164	3.417	4.075		4.157	0.230	3.696	4.618	
low		0.802	0.077	0.648	0.957		1.357	0.099	1.159	1.554		2.314	0.169	1.977	2.652		2.674	0.232	2.210	3.139	
Belowground biomass																					
Origin		fit	se	lower	upper	*															.
DE		0.368	0.025	0.318	0.418																
NZ		0.289	0.030	0.230	0.349																
Water		fit	se	lower	upper	***	fit	se	lower	upper	***	fit	se	lower	upper	***	fit	se	lower	upper	***
well		0.446	0.026	0.394	0.499		1.245	0.067	1.111	1.379		1.935	0.154	1.627	2.243		3.849	0.199	3.450	4.248	
low		0.225	0.026	0.173	0.278		0.404	0.067	0.271	0.538		0.571	0.156	0.259	0.882		0.795	0.202	0.391	1.200	
Origin x Water						.	fit	se	lower	upper	**						fit	se	lower	upper	**
DE	well						1.375	0.076	1.223	1.527							4.505	0.269	3.966	5.043	
NZ	well						1.078	0.082	0.915	1.241							2.997	0.300	2.395	3.598	
DE	low						0.377	0.077	0.222	0.531							0.705	0.267	0.171	1.239	
NZ	low						0.440	0.084	0.272	0.607							0.913	0.312	0.290	1.537	
UV-B x Water							fit	se	lower	upper	*										
- UV-B	well						1.425	0.095	1.236	1.615											
+ UV-B	well						1.070	0.094	0.882	1.259											
- UV-B	low						0.441	0.094	0.253	0.629											
+ UV-B	low						0.368	0.094	0.180	0.557											
Shoot:mass ratio																					
Water		fit	se	lower	upper	*	fit	se	lower	upper	***	fit	se	lower	upper	***	fit	se	lower	upper	***
well		0.753	0.008	0.737	0.769		0.664	0.013	0.639	0.689		0.667	0.017	0.633	0.701		0.530	0.013	0.504	0.556	
low		0.779	0.008	0.762	0.795		0.774	0.012	0.749	0.798		0.800	0.017	0.767	0.834		0.783	0.013	0.757	0.809	
Origin x Water																	fit	se	lower	upper	*
DE	well																0.510	0.017	0.475	0.545	
NZ	well																0.557	0.019	0.518	0.596	
DE	low																0.784	0.017	0.749	0.819	
NZ	low																0.781	0.020	0.741	0.821	
Root dry matter content																					
Origin							fit	se	lower	upper	*										
DE							12.740	0.323	12.094	13.386											
NZ							11.616	0.374	10.867	12.364											
Water		fit	se	lower	upper	***						fit	se	lower	upper	*					
well		10.501	0.559	9.383	11.619							23.938	1.891	20.154	27.721						
low		13.693	0.559	12.575	14.811							29.064	1.948	25.166	32.961						
UV-B x Water							fit	se	lower	upper	*										
- UV-B	well						12.487	0.441	11.607	13.368											
+ UV-B	well						11.407	0.431	10.545	12.269											
- UV-B	low						12.174	0.429	11.317	13.032											
+ UV-B	low						12.915	0.431	12.055	13.776											
Leaf dry matter content																					
Origin		fit	se	lower	upper	*	fit	se	lower	upper	***										
DE		17.221	0.356	16.508	17.934		19.461	0.321	18.820	20.102											
NZ		15.867	0.436	14.994	16.741		17.391	0.376	16.639	18.143											
Water		fit	se	lower	upper	***	fit	se	lower	upper	***	fit	se	lower	upper	***	fit	se	lower	upper	**
well		14.668	0.376	13.916	15.420		19.510	0.304	18.902	20.117		28.482	0.719	27.044	29.921		26.506	1.056	24.393	28.619	
low		18.744	0.381	17.980	19.507		17.631	0.299	17.032	18.230		32.743	0.740	31.262	34.224		30.190	1.069	28.053	32.328	
Specific leaf area																					
Water							fit	se	lower	upper	*	fit	se	lower	upper	*					
well							16.499	1.289	13.922	19.076		13.976	0.841	12.293	15.658						
low							19.844	1.276	17.294	22.394		11.979	0.848	10.282	13.675						
Origin x Water																	fit	se	lower	upper	**
DE	well																18.610	0.930	16.749	20.471	
NZ	well																17.105	1.045	15.456	19.634	
DE	low																17.569	0.924	15.720	19.417	
NZ	low																21.314	1.075	19.165	23.464	

		1st harvest (3 weeks)					2nd harvest (6 weeks)					3rd harvest (9 weeks)					4th harvest (12 weeks)				
Leaf number																					
**UV-B**							**fit**	**se**	**lower**	**upper**	**										
- UV-B							15.187	0.317	14.553	15.821											
+ UV-B							16.062	0.314	15.434	16.691											
**Water**		**fit**	**se**	**lower**	**upper**	***	**fit**	**se**	**lower**	**upper**	***	**fit**	**se**	**lower**	**upper**	***	**fit**	**se**	**lower**	**upper**	***
well		11.811	0.219	11.373	12.249		16.888	0.317	16.254	17.522		19.198	0.442	18.314	20.082		23.370	0.560	22.250	24.491	
low		10.689	0.219	10.251	11.127		14.409	0.315	13.780	15.038		16.717	0.451	15.816	17.619		18.983	0.563	17.857	20.109	
**Origin x UV-B**												**fit**	**se**	**lower**	**upper**	*					
DE	- UV-B											18.732	0.605	17.522	19.942						
NZ	- UV-B											17.342	0.716	15.908	18.775						
DE	+ UV-B											17.702	0.603	16.496	18.909						
NZ	+ UV-B											18.009	0.711	16.587	19.432						
**Origin x Water**																	**fit**	**se**	**lower**	**upper**	**
DE	well																24.565	0.750	23.065	26.065	
NZ	well																21.817	0.833	20.152	23.483	
DE	low																19.114	0.745	17.624	20.604	
NZ	low																18.814	0.846	17.121	20.506	
Proportion of dead leaves																					
**Water**																	**fit**	**se**	**lower**	**upper**	*
well																	29.530	1.652	26.226	32.834	
low																	33.391	1.664	30.062	36.719	
**Origin x UV-B**		**fit**	**se**	**lower**	**upper**	**															
DE	- UV-B	22.798	3.341	16.112	29.484																
NZ	- UV-B	17.054	3.690	9.669	24.438																
DE	+ UV-B	17.674	3.335	11.000	24.348																
NZ	+ UV-B	26.486	3.734	19.013	33.958																
**Origin x Water**							**fit**	**se**	**lower**	**upper**	*										
DE	well						26.070	2.338	21.397	30.743											
NZ	well						29.248	2.665	23.921	34.575											
DE	low						27.735	2.394	22.949	32.521											
NZ	low						22.635	2.835	16.968	28.302											
Leaf length																					
**UV-B**		**fit**	**se**	**lower**	**upper**	*						**fit**	**se**	**lower**	**upper**	*					
- UV-B		15.91784	0.447	15.02325	16.81244							14.251	0.282	13.687	14.815						
+ UV-B		10.80275	0.447	9.908	11.69734							13.393	0.282	12.829	13.956						
**Water**		**fit**	**se**	**lower**	**upper**	*	**fit**	**se**	**lower**	**upper**	***	**fit**	**se**	**lower**	**upper**	***	**fit**	**se**	**lower**	**upper**	***
well		13.962	0.399	13.163	14.761		16.905	0.273	16.360	17.451		15.733	0.278	15.177	16.289		15.319	0.282	14.756	15.883	
low		12.759	0.399	11.960	13.557		12.544	0.269	12.007	13.082		11.795	0.286	11.223	12.367		12.018	0.285	11.448	12.587	
**Origin x Water**							**fit**	**se**	**lower**	**upper**	**						**fit**	**se**	**lower**	**upper**	*
DE	well						16.442	0.380	15.682	17.202							15.133	0.386	14.360	15.905	
NZ	well						17.504	0.434	16.635	18.372							15.562	0.433	14.695	16.429	
DE	low						13.031	0.389	12.254	13.809							12.510	0.384	11.743	13.278	
NZ	low						11.916	0.460	10.995	12.836							11.377	0.445	10.487	12.267	
Leaf width																					
**Origin**		**fit**	**se**	**lower**	**upper**	*	**fit**	**se**	**lower**	**upper**	*										
DE		6.208	0.239	5.730	6.686		6.575	0.155	6.266	6.885											
NZ		5.649	0.261	5.128	6.171		6.008	0.179	5.650	6.367											
**Water**		**fit**	**se**	**lower**	**upper**	***	**fit**	**se**	**lower**	**upper**	***	**fit**	**se**	**lower**	**upper**	***	**fit**	**se**	**lower**	**upper**	***
well		6.440	0.239	5.962	6.918		7.208	0.124	6.960	7.456		6.862	0.187	6.489	7.236		8.467	0.400	7.667	9.267	
low		5.516	0.239	5.038	5.993		5.472	0.123	5.226	5.718		5.605	0.190	5.225	5.985		6.310	0.405	5.499	7.121	
**Origin x Water**												**fit**	**se**	**lower**	**upper**	*					
DE	well											6.877	0.249	6.378	7.376						
NZ	well											6.841	0.287	6.267	7.416						
DE	low											5.910	0.248	5.414	6.406						
NZ	low											5.170	0.301	4.567	5.773						
Rosette area																					
**UV-B**		**fit**	**se**	**lower**	**upper**	*															
- UV-B		446.187	28.721	388.716	503.659																
+ UV-B		206.260	28.721	148.788	263.731																
**Water**							**fit**	**se**	**lower**	**upper**	***	**fit**	**se**	**lower**	**upper**	***	**fit**	**se**	**lower**	**upper**	***
well							556.943	17.94022	521.081	592.805		642.2073	20.5646	601.0577	683.3568		451.381	20.759	409.857	492.906	
low							303.375	17.71059	267.972	338.778		367.8283	21.1644	325.4784	410.1783		269.643	20.956	227.724	311.561	
PSII efficiency (Y)																					
**UV-B**		**fit**	**se**	**lower**	**upper**	**															
- UV-B		0.825	0.002	0.821	0.829																
+ UV-B		0.834	0.002	0.830	0.838																
**Water**		**fit**	**se**	**lower**	**upper**	**	**fit**	**se**	**lower**	**upper**	***	**fit**	**se**	**lower**	**upper**	***	**fit**	**se**	**lower**	**upper**	**
well		0.825	0.002	0.821	0.830		0.814	0.003	0.809	0.820		0.802	0.004	0.795	0.809		0.795	0.004	0.788	0.802	
low		0.834	0.002	0.830	0.838		0.825	0.003	0.819	0.830		0.818	0.004	0.811	0.825		0.803	0.004	0.796	0.810	
**UV-B x Water**							**fit**	**se**	**lower**	**upper**	**										
- UV-B	well						0.805	0.004	0.798	0.812											
+ UV-B	well						0.823	0.004	0.816	0.830											
- UV-B	low						0.824	0.004	0.817	0.831											
+ UV-B	low						0.826	0.004	0.819	0.833											
Min. chlorophyll fluorescence (F_0)																					
**Origin**							**fit**	**se**	**lower**	**upper**	*	**fit**	**se**	**lower**	**upper**	*	**fit**	**se**	**lower**	**upper**	**
DE							394.810	8.499	377.822	411.799		378.2706	11.9998	354.2591	402.2822		354.963	15.473	324.012	385.914	
NZ							427.996	9.802	408.401	447.590		414.4455	13.7182	386.9955	441.8955		388.209	15.966	356.272	420.147	
**Water**		**fit**	**se**	**lower**	**upper**	**	**fit**	**se**	**lower**	**upper**	*						**fit**				***
well		418.098	7.248	403.594	432.601		418.183	7.363	403.465	432.901							354.120	15.267	323.581	384.659	
low		389.344	7.248	374.840	403.847		400.663	7.290	386.090	415.236							385.165	15.311	354.538	415.793	
**Origin x UV-B**		**fit**	**se**	**lower**	**upper**	*															
DE	- UV-B	405.511	9.782	385.937	425.085																
NZ	- UV-B	426.437	11.016	404.394	448.480																
DE	+ UV-B	399.024	9.761	379.493	418.555																
NZ	+ UV-B	385.156	11.170	362.805	407.507																
**UV-B x Water**							**fit**	**se**	**lower**	**upper**	**										
- UV-B	well						448.308	9.873	428.571	468.045											
+ UV-B	well						388.895	9.688	369.529	408.262											
- UV-B	low						405.610	9.635	386.351	424.870											
+ UV-B	low						395.853	9.676	376.511	415.194											
Max. chlorophyll fluorescence (F_m)																					
**Water**												**fit**	**se**	**lower**	**upper**	**	**fit**	**se**	**lower**	**upper**	***
well												1995.441	78.317	1838.728	2152.153		1734.201	85.130	1563.916	1904.486	
low												2154.388	78.750	1996.809	2311.966		1965.041	85.261	1794.493	2135.588	
**Origin x UV-B**		**fit**	**se**	**lower**	**upper**	*															
DE	- UV-B	2334.922	45.296	2244.285	2425.560																
NZ	- UV-B	2431.805	53.113	2325.527	2538.083																
DE	+ UV-B	2431.648	45.158	2341.288	2522.008																
NZ	+ UV-B	2293.737	54.066	2185.550	2401.923																

Table S2: Effect directions of repeated measures analysis. Predicted values ('fit'), standard error ('SE') and confidence interval ('lower' and 'upper' limits) are given for all significant main and twofold interaction effects of 'Origin', 'UV-B', 'Water' and 'Time' in the repeated measures analysis. Significance levels of effects are indicated by asterisks (*P < 0.05; **P < 0.01; ***P < 0.001).

leaf number						
**Origin**		**fit**	**se**	**lower**	**upper**	**
DE		9.942	0.246	9.459	10.426	
NZ		8.755	0.276	8.215	9.296	
**Water**		**fit**	**se**	**lower**	**upper**	***
well		10.185	0.203	9.787	10.583	
low		8.675	0.203	8.276	9.074	
**Time**		**fit**	**se**	**lower**	**upper**	***
0		6.924	0.191	6.549	7.298	
20		8.797	0.189	8.427	9.167	
40		10.670	0.198	10.282	11.059	
60		12.544	0.218	12.117	12.971	
80		14.417	0.245	13.936	14.898	
**UV-B x Time**		**fit**	**se**	**lower**	**upper**	*
- UV-B	0	6.938	0.217	6.513	7.363	
+ UV-B	0	6.910	0.216	6.486	7.333	
- UV-B	20	8.691	0.213	8.273	9.108	
+ UV-B	20	8.902	0.212	8.486	9.318	
- UV-B	40	10.443	0.229	9.993	10.893	
+ UV-B	40	10.895	0.229	10.447	11.343	
- UV-B	60	12.196	0.263	11.680	12.711	
+ UV-B	60	12.887	0.262	12.374	13.400	
- UV-B	80	13.948	0.308	13.345	14.552	
+ UV-B	80	14.880	0.306	14.280	15.480	
**Water x Time**		**fit**	**se**	**lower**	**upper**	***
well	0	7.292	0.204	6.892	7.691	
low	0	6.548	0.204	6.149	6.948	
well	20	9.448	0.199	9.057	9.839	
low	20	8.133	0.200	7.742	8.525	
well	40	11.604	0.217	11.179	12.029	
low	40	9.718	0.217	9.292	10.144	
well	60	13.760	0.251	13.267	14.252	
low	60	11.303	0.253	10.808	11.798	
well	80	15.915	0.297	15.333	16.498	
low	80	12.888	0.299	12.301	13.475	

leaf length						
**Origin**		**fit**	**se**	**lower**	**upper**	***
DE		12.959	0.252	12.465	13.454	
NZ		11.590	0.278	11.045	12.137	
**Water**		**fit**	**se**	**lower**	**upper**	***
well		14.091	0.224	13.652	14.531	
low		10.628	0.225	10.188	11.069	
**Time**		**fit**	**se**	**lower**	**upper**	***
0		9.376	0.235	8.916	9.836	
20		11.614	0.206	11.209	12.018	
40		13.851	0.222	13.416	14.285	
60		16.088	0.273	15.553	16.622	
80		18.325	0.344	17.649	19.001	
**Origin x Time**		**fit**	**se**	**lower**	**upper**	***
DE	0	10.658	0.291	10.087	11.230	
NZ	0	7.643	0.327	7.002	8.284	
DE	20	12.373	0.252	11.880	12.867	
NZ	20	10.586	0.278	10.040	11.132	
DE	40	14.089	0.273	13.553	14.625	
NZ	40	13.529	0.304	12.932	14.125	
DE	60	15.804	0.345	15.128	16.480	
NZ	60	16.472	0.390	15.707	17.237	
DE	80	17.519	0.443	16.651	18.387	
NZ	80	19.415	0.506	18.422	20.408	
**Water x Time**		**fit**	**se**	**lower**	**upper**	***
well	0	10.332	0.273	9.796	10.868	
low	0	8.401	0.274	7.863	8.939	
well	20	13.134	0.224	12.695	13.573	
low	20	10.061	0.224	9.622	10.501	
well	40	15.937	0.251	15.445	16.428	
low	40	11.722	0.251	11.227	12.216	
well	60	18.739	0.336	18.080	19.398	
low	60	13.382	0.339	12.717	14.047	
well	80	21.542	0.448	20.664	22.420	
low	80	15.042	0.452	14.155	15.929	

leaf width						
**Origin**		**fit**	**se**	**lower**	**upper**	***
DE		6.076	0.587	4.924	7.227	
NZ		4.956	0.615	3.750	6.162	
**Water**		**fit**	**se**	**lower**	**upper**	***
well		6.231	0.536	5.180	7.283	
low		4.955	0.536	3.903	6.006	
**Time**		**fit**	**se**	**lower**	**upper**	***
0		4.465	0.537	3.413	5.518	
20		5.311	0.535	4.262	6.360	
40		6.156	0.535	5.106	7.206	
60		7.002	0.538	5.947	8.056	
80		7.847	0.542	6.784	8.910	
**UV-B x Time**		**fit**	**se**	**lower**	**upper**	***
- UV-B	0	4.626	0.710	3.234	6.018	
+ UV-B	0	4.307	0.710	2.916	5.699	
- UV-B	20	5.333	0.707	3.946	6.720	
+ UV-B	20	5.289	0.707	3.902	6.676	
- UV-B	40	6.040	0.708	4.651	7.428	
+ UV-B	40	6.271	0.708	4.883	7.659	
- UV-B	60	6.746	0.711	5.351	8.142	
+ UV-B	60	7.253	0.711	5.858	8.648	
- UV-B	80	7.453	0.718	6.045	8.861	
+ UV-B	80	8.235	0.718	6.827	9.643	
**Origin x Time**		**fit**	**se**	**lower**	**upper**	***
DE	0	5.082	0.590	3.925	6.238	
NZ	0	3.632	0.618	2.419	4.845	
DE	20	5.823	0.587	4.671	6.974	
NZ	20	4.619	0.615	3.412	5.825	
DE	40	6.564	0.588	5.411	7.716	
NZ	40	5.606	0.616	4.398	6.813	
DE	60	7.304	0.592	6.144	8.465	
NZ	60	6.592	0.621	5.375	7.810	
DE	80	8.045	0.598	6.872	9.219	
NZ	80	7.579	0.629	6.345	8.813	
**Water x Time**		**fit**	**se**	**lower**	**upper**	***
well	0	4.821	0.539	3.763	5.879	
low	0	4.103	0.539	3.045	5.161	
well	20	5.872	0.536	4.821	6.924	
low	20	4.738	0.536	3.686	5.790	
well	40	6.924	0.537	5.870	7.977	
low	40	5.373	0.537	4.320	6.426	
well	60	7.975	0.542	6.913	9.037	
low	60	6.008	0.542	4.946	7.071	
well	80	9.027	0.550	7.948	10.105	
low	80	6.643	0.550	5.564	7.723	

proportion of dead leaves						
**Water**		**fit**	**se**	**lower**	**upper**	***
well		2.650	0.133	2.389	2.911	
low		2.552	0.133	2.290	2.813	
**Time**		**fit**	**se**	**lower**	**upper**	***
0		0.309	0.123	0.067	0.551	
20		2.017	0.123	1.775	2.259	
40		3.725	0.131	3.468	3.983	
60		5.434	0.146	5.147	5.720	
80		7.142	0.166	6.816	7.468	
**Water x Time**		**fit**	**se**	**lower**	**upper**	***
well	0	0.163	0.130	-0.091	0.418	
low	0	0.457	0.130	0.202	0.712	
well	20	2.016	0.130	1.762	2.27	
low	20	2.018	0.130	1.764	2.272	
well	40	3.869	0.144	3.586	4.152	
low	40	3.579	0.144	3.295	3.862	
well	60	5.722	0.170	5.389	6.056	
low	60	5.139	0.171	4.803	5.475	
well	80	7.575	0.203	7.177	7.973	
low	80	6.700	0.205	6.298	7.102	

rosette area

Effect	Level	fit	se	lower	upper	
**Origin**		**fit**	**se**	**lower**	**upper**	***
DE		355.742	18.738	318.992	392.493	
NZ		285.245	20.375	245.285	325.204	
**Water**		**fit**	**se**	**lower**	**upper**	***
well		420.729	16.101	389.150	452.307	
low		228.859	16.112	197.260	260.459	
**Time**		**fit**	**se**	**lower**	**upper**	***
0		158.289	16.121	126.671	189.907	
20		283.143	15.665	252.419	313.866	
40		408.000	15.941	376.731	439.260	
60		532.849	16.914	499.677	566.021	
80		657.702	18.473	621.471	693.932	
**Origin x Time**		**fit**	**se**	**lower**	**upper**	***
DE	0	206.761	19.378	168.756	244.766	
NZ	0	92.742	21.184	51.196	134.289	
DE	20	317.822	18.720	281.107	354.537	
NZ	20	236.247	20.355	196.325	276.169	
DE	40	428.882	19.125	391.375	466.390	
NZ	40	379.751	20.848	338.863	420.639	
DE	60	539.943	20.529	499.681	580.205	
NZ	60	523.255	22.575	478.980	567.530	
DE	80	651.004	22.748	606.389	695.619	
NZ	80	666.759	25.285	617.170	716.349	
**Water x Time**		**fit**	**se**	**lower**	**upper**	***
well	0	193.729	16.949	160.488	226.970	
low	0	122.120	16.970	88.837	155.402	
well	20	362.950	16.079	331.415	394.485	
low	20	201.691	16.087	170.140	233.241	
well	40	532.171	16.605	499.605	564.738	
low	40	281.261	16.632	248.641	313.881	
well	60	701.392	18.407	665.292	737.492	
low	60	360.832	18.480	324.588	397.076	
well	80	870.613	21.151	829.111	912.116	
low	80	440.403	21.294	398.640	482.165	

PSII efficiency (Y)

Effect	Level	fit	se	lower	upper	
**Origin**		**fit**	**se**	**lower**	**upper**	**
DE		0.822	0.002	0.818	0.825	
NZ		0.814	0.002	0.811	0.818	
**UV-B x Time**		**fit**	**se**	**lower**	**upper**	*
- UV-B	0	0.817	0.002	0.814	0.820	
+ UV-B	0	0.819	0.002	0.816	0.822	
- UV-B	20	0.816	0.001	0.813	0.819	
+ UV-B	20	0.821	0.001	0.818	0.823	
- UV-B	40	0.815	0.002	0.812	0.818	
+ UV-B	40	0.822	0.002	0.819	0.825	
- UV-B	60	0.814	0.002	0.810	0.818	
+ UV-B	60	0.824	0.002	0.820	0.827	
- UV-B	80	0.813	0.002	0.808	0.818	
+ UV-B	80	0.825	0.002	0.820	0.830	
**Water x Time**		**fit**	**se**	**lower**	**upper**	***
well	0	0.818	0.002	0.815	0.821	
low	0	0.819	0.002	0.816	0.822	
well	20	0.816	0.001	0.813	0.819	
low	20	0.821	0.001	0.818	0.824	
well	40	0.814	0.002	0.811	0.817	
low	40	0.823	0.002	0.820	0.826	
well	60	0.813	0.002	0.809	0.816	
low	60	0.825	0.002	0.822	0.829	
well	80	0.811	0.002	0.806	0.816	
low	80	0.828	0.002	0.823	0.832	

min. chlorophyll fluorescence

Effect	Level	fit	se	lower	upper	
**Origin**		**fit**	**se**	**lower**	**upper**	**
DE		407.458	5.431	396.808	418.108	
NZ		434.476	5.992	422.724	446.228	
**Water**		**fit**	**se**	**lower**	**upper**	*
well		423.606	4.556	414.671	432.542	
low		413.801	4.561	404.857	422.745	
**Time**		**fit**	**se**	**lower**	**upper**	***
0		439.693	4.578	430.715	448.670	
20		419.798	4.385	411.199	428.398	
40		399.904	4.520	391.038	408.769	
60		380.009	4.957	370.287	389.731	
80		360.115	5.626	349.081	371.148	
**UV-B x Time**		**fit**	**se**	**lower**	**upper**	***
- UV-B	0	438.390	5.514	427.577	449.203	
+ UV-B	0	440.976	5.500	430.190	451.762	
- UV-B	20	423.377	5.189	413.200	433.554	
+ UV-B	20	416.272	5.179	406.115	426.430	
- UV-B	40	408.364	5.420	397.734	418.994	
+ UV-B	40	391.568	5.405	380.967	402.168	
- UV-B	60	393.351	6.144	381.302	405.401	
+ UV-B	60	366.864	6.117	354.867	378.861	
- UV-B	80	378.338	7.214	364.190	392.486	
+ UV-B	80	342.159	7.172	328.094	356.225	

max. chlorophyll fluorescence

Effect	Level	fit	se	lower	upper	
**Water**		**fit**	**se**	**lower**	**upper**	*
well		2303.269	89.985	2126.794	2479.743	
low		2308.768	89.989	2132.285	2485.250	
**Time**		**fit**	**se**	**lower**	**upper**	***
0		2416.228	89.985	2239.755	2592.701	
20		2311.483	89.860	2135.254	2487.713	
40		2206.739	90.005	2030.227	2383.251	
60		2101.995	90.416	1924.676	2279.314	
80		1997.250	91.091	1818.608	2175.892	
**UV-B x Time**		**fit**	**se**	**lower**	**upper**	**
- UV-B	0	2393.358	127.066	2144.163	2642.554	
+ UV-B	0	2438.760	127.059	2189.579	2687.942	
- UV-B	20	2304.274	126.889	2055.426	2553.123	
+ UV-B	20	2318.586	126.885	2069.746	2567.426	
- UV-B	40	2215.191	127.096	1965.936	2464.445	
+ UV-B	40	2198.412	127.087	1949.175	2447.648	
- UV-B	60	2126.107	127.684	1875.699	2376.515	
+ UV-B	60	2078.237	127.664	1827.869	2328.606	
- UV-B	80	2037.023	128.649	1784.724	2289.323	
+ UV-B	80	1958.063	128.611	1705.838	2210.288	
**Origin x Time**		**fit**	**se**	**lower**	**upper**	**
DE	0	2405.517	90.370	2228.289	2582.746	
NZ	0	2431.134	90.679	2253.298	2608.969	
DE	20	2288.722	90.162	2111.902	2465.543	
NZ	20	2343.161	90.376	2165.920	2520.401	
DE	40	2171.927	90.415	1994.610	2349.244	
NZ	40	2255.188	90.706	2077.299	2433.076	
DE	60	2055.132	91.126	1876.421	2233.842	
NZ	60	2167.215	91.664	1987.449	2346.981	
DE	80	1938.336	92.283	1757.356	2119.317	
NZ	80	2079.242	93.229	1896.407	2262.077	
**Water x Time**		**fit**	**se**	**lower**	**upper**	***
well	0	2434.017	90.228	2257.067	2610.968	
low	0	2398.065	90.237	2221.096	2575.034	
well	20	2309.785	89.984	2133.313	2486.256	
low	20	2313.218	89.987	2136.739	2489.697	
well	40	2185.552	90.267	2008.526	2362.578	
low	40	2228.371	90.281	2051.316	2405.426	
well	60	2061.319	91.072	1882.713	2239.926	
low	60	2143.524	91.113	1964.838	2322.210	
well	80	1937.087	92.387	1755.902	2118.272	
low	80	2058.677	92.469	1877.333	2240.022	
**Origin x UV-B**		**fit**	**se**	**lower**	**upper**	*
DE	- UV-B	2264.442	127.168	2015.046	2513.838	
NZ	- UV-B	2348.535	127.390	2098.705	2598.365	
DE	+ UV-B	2300.483	127.165	2051.094	2549.872	
NZ	+ UV-B	2328.705	127.369	2078.916	2578.495	

Table S3: Fixed-effect results of the harvest data analysis. ‚UV-B' and 'Water' depict the effect of treatments, 'Origin' refers to the effect of German vs. New Zealand provenance. Degrees of freedom (dfN = numerator, dfD = denominator), F statistics (F), and significance values (P) are provided. Values in boldface type indicate significant P values (*P < 0.05; **P < 0.01; ***P < 0.001) and values in italic typeface indicate marginal effects. (P < 0.1).

		1st harvest (3 weeks)			2nd harvest (6 weeks)			3rd harvest (9 weeks)			4th harvest (12 weeks)		
Variable / Source	df_N	df_D	F	p	df_D	F	p	df_D	F	p	df_D	F	p
Leaf number													
Origin	1	16.5	0.366	0.554	20.7	0.176	0.680	20.8	0.390	0.539	17.9	2.131	0.162
UV-B	1	44.3	0.050	0.825	46.1	8.537	0.005 **	44.0	0.200	0.657	1.6	0.150	0.743
Water	1	45.3	15.466	<0.001 ***	46.1	60.636	<0.001 ***	44.0	40.880	<0.001 ***	44.6	111.702	<0.001 ***
Initial leaf number (Covariate)	1	49.5	52.319	<0.001 ***	52.4	12.779	<0.001 ***	53.2	15.095	<0.001 ***	55.9	24.796	<0.001 ***
Origin x UV-B	1	44.2	0.289	0.594	47.0	0.959	0.332	43.9	4.556	0.038 *	42.2	0.078	0.782
Origin x Water	1	44.3	0.025	0.875	47.0	0.655	0.422	43.9	0.457	0.503	44.2	9.566	0.003 **
UV-B x Water	1	44.9	0.225	0.637	47.1	0.000	0.993	44.0	0.116	0.735	44.5	0.549	0.463
Origin x UV-B x Water	1	44.1	1.865	0.179	46.5	0.244	0.624	43.9	0.219	0.642	45.5	0.244	0.623
Proportion of dead leaves													
Origin	1	57.0	0.293	0.590	18.8	0.082	0.778	18.7	0.43487	0.518	17.8	0.885	0.360
UV-B	1	1.5	0.265	0.671	45.1	2.358	0.132	1.7	0.0033	0.960	44.2	0.382	0.540
Water	1	58.0	3.368	0.072 .	45.2	1.677	0.202	41.1	0.14482	0.706	44.3	6.813	0.012 *
Initial leaf number (Covariate)	1	57.1	19.180	<0.001 ***	41.0	0.535	0.469	55.0	3.1216	0.083 .	59.0	0.214	0.645
Origin x UV-B	1	57.0	7.745	0.007 **	46.1	0.003	0.957	38.9	2.73949	0.106	44.1	0.136	0.714
Origin x Water	1	41.3	1.283	0.264	46.2	4.581	0.038 *	41.1	0.00291	0.957	44.2	0.026	0.872
UV-B x Water	1	58.0	0.045	0.832	46.2	0.505	0.481	41.2	0.00179	0.966	44.2	1.002	0.322
Origin x UV-B x Water	1	41.0	0.997	0.324	45.6	1.426	0.239	41.2	0.81822	0.371	45.4	0.726	0.399
Leaf length													
Origin	1	16.0	3.161	0.094 .	62.0	0.003	0.953	23.2	0.057	0.813	18.9	0.447	0.512
UV-B	1	1.4	67.862	0.039 *	62.0	0.702	0.405	44.4	6.697	0.013 *	45.2	1.672	0.203
Water	1	43.9	6.304	0.016 *	62.0	135.225	<0.001 ***	44.5	126.87	<0.001 ***	45.2	134.029	<0.001 ***
Initial leaf number (Covariate)	1	42.1	2.924	0.095 .	62.0	1.510	0.224	58.9	0.019	0.891	59.9	0.404	0.527
Origin x UV-B	1	41.8	0.504	0.482	62.0	0.168	0.683	44.4	1.412	0.241	45.1	0.664	0.419
Origin x Water	1	26.1	0.000	0.987	62.0	7.756	0.007 **	44.4	2.188	0.146	45.2	7.134	0.010 *
UV-B x Water	1	43.5	0.287	0.595	62.0	1.845	0.179	44.4	0.339	0.562	45.1	0.897	0.349
Origin x UV-B x Water	1	25.8	0.930	0.344	62.0	0.000	0.985	44.4	0.286	0.595	46.6	0.975	0.329
Leaf width													
Origin	1	17.1	5.067	0.038 *	21.8	4.894	0.038 *	21.5	1.218	0.282	19.4	3.744	0.068 .
UV-B	1	1.7	13.612	0.085 .	47.1	0.198	0.659	1.5	0.544	0.560	1.8	19.048	0.058 .
Water	1	44.3	21.783	<0.001 ***	47.1	191.932	<0.001 ***	40.2	62.907	<0.001 ***	46.8	18.847	<0.001 ***
Initial leaf number (Covariate)	1	45.9	0.209	0.649	51.6	1.125	0.294	54.0	1.446	0.234	46.1	0.541	0.466
Origin x UV-B	1	42.8	0.060	0.808	48.0	0.118	0.733	42.3	2.027	0.162	44.7	1.607	0.212
Origin x Water	1	42.6	1.121	0.296	48.0	0.438	0.511	40.1	4.486	0.040 *	46.4	0.002	0.963
UV-B x Water	1	43.9	1.148	0.290	48.1	0.057	0.812	39.1	1.643	0.207	46.5	4.007	0.051 .
Origin x UV-B x Water	1	42.5	0.551	0.462	47.5	2.187	0.146	38.7	0.627	0.433	48.6	0.443	0.509
Rosette area													
Origin	1	16.5	0.717	0.409	17.5	0.538	0.473	17.2	0.384	0.543	19.8	3.123	0.093 .
UV-B	1	1.5	38.547	0.049 *	45.1	1.458	0.234	1.2	0.479	0.597	1.9	6.899	0.127
Water	1	44.0	3.528	0.067 .	45.2	116.426	<0.001 ***	30.0	107.454	<0.001 ***	46.1	78.247	<0.001 ***
Initial leaf number (Covariate)	1	47.8	0.013	0.909	32.1	0.379	0.542	57.4	2.388	0.128	54.4	0.011	0.917
Origin x UV-B	1	42.2	0.419	0.521	46.0	1.412	0.241	36.8	1.420	0.241	44.3	2.222	0.143
Origin x Water	1	32.7	0.741	0.396	46.2	0.148	0.703	30.3	2.909	0.098 .	45.6	0.899	0.348
UV-B x Water	1	43.7	1.428	0.238	46.1	1.192	0.281	28.9	1.020	0.321	45.9	2.367	0.131
Origin x UV-B x Water	1	32.4	0.000	0.999	45.5	0.001	0.971	28.6	0.371	0.547	47.9	0.005	0.943
PSII efficiency (Y)													
Origin	1	59.0	0.752	0.389	20.6	3.504	0.075 .	22.0	1.128	0.300	18.3	2.246	0.151
UV-B	1	59.0	9.160	0.004 **	1.8	8.962	0.107	1.7	0.068	0.823	1.7	17.498	0.068 .
Water	1	59.0	8.725	0.005 **	45.4	12.675	0.001 ***	43.3	23.580	<0.001 ***	44.9	11.329	0.002 **
Initial leaf number (Covariate)	1	59.0	9.242	0.004 **	43.5	0.969	0.330	58.5	3.394	0.071 .	56.8	1.561	0.217
Origin x UV-B	1	59.0	0.233	0.631	45.3	0.004	0.953	41.2	0.129	0.721	42.7	2.381	0.130
Origin x Water	1	59.0	0.038	0.847	44.9	0.043	0.827	43.3	0.198	0.659	44.6	0.028	0.869
UV-B x Water	1	59.0	1.451	0.233	46.3	8.507	0.005 **	43.3	1.444	0.236	44.8	1.565	0.217
Origin x UV-B x Water	1	59.0	0.001	0.970	44.2	2.289	0.137	43.3	0.095	0.760	45.9	0.674	0.416
Min. chlorophyll fluorescence (F_0)													
Origin	1	57.0	0.148	0.702	20.2	5.901	0.025 *	24.1	4.960	0.036 *	18.9	9.213	0.007 **
UV-B	1	1.4	4.054	0.231	1.9	13.014	0.073 .	1.7	1.562	0.358	2.0	0.529	0.544
Water	1	58.2	10.856	0.002 **	46.1	4.921	0.032 *	44.5	0.367	0.548	44.4	12.962	0.001 ***
Initial leaf number (Covariate)	1	57.2	22.879	<0.001 ***	38.5	0.082	0.776	58.3	0.323	0.572	51.0	0.340	0.562
Origin x UV-B	1	57.0	4.230	0.044 *	46.1	0.208	0.650	43.1	0.961	0.333	43.5	1.740	0.194
Origin x Water	1	32.5	0.075	0.787	45.8	0.540	0.466	44.5	1.030	0.316	43.8	0.802	0.375
UV-B x Water	1	58.2	2.272	0.137	47.0	9.555	0.003 **	44.3	0.071	0.791	44.1	0.230	0.634
Origin x UV-B x Water	1	32.1	0.321	0.575	45.2	1.390	0.245	44.2	1.160	0.287	46.2	2.430	0.126
Max. chlorophyll fluorescence (F_m)													
Origin	1	57.3	0.167	0.684	20.5	0.930	0.346	21.9	3.334	0.081 .	19.4	1.546	0.229
UV-B	1	1.2	0.165	0.746	1.9	1.486	0.353	1.9	0.434	0.581	2.0	1.926	0.300
Water	1	58.8	0.964	0.330	46.2	1.120	0.295	42.2	9.100	0.004 **	44.0	46.199	<0.001 ***
Initial leaf number (Covariate)	1	57.3	6.320	0.015 *	37.1	0.501	0.483	56.9	0.262	0.610	57.0	0.320	0.574
Origin x UV-B	1	57.3	6.428	0.014 *	46.6	0.189	0.666	41.1	0.791	0.379	43.4	0.126	0.725
Origin x Water	1	16.8	0.745	0.400	46.4	0.336	0.565	42.3	0.177	0.676	43.5	0.691	0.411
UV-B x Water	1	58.8	0.327	0.570	47.0	0.901	0.347	42.5	0.278	0.601	43.9	0.020	0.888
Origin x UV-B x Water	1	16.5	0.444	0.515	45.8	0.009	0.926	42.6	1.098	0.301	45.7	1.404	0.242

Table S4: Location and sampling information of native (German) and exotic (New Zealand) populations included in the experiment

Origin	*Latitude*	*Longitude*	*Locality*	*Collection year*	*n*
Germany (native)	51.48431	11.90097	Nietleben	2012	16
	51.62067	11.74124	Friedenburg	2012	16
	51.52282	12.00856	Tornau	2012	15
	51.51693	12.01146	Mötzlich	2012	16
	51.74834	11.02791	Thale	2012	16
	51.78530	11.15421	Quedlinburg I	2012	16
	51.77611	11.13432	Quedlinburg II	2012	16
	51.73221	11.21278	Ballenstedt	2012	16
	51.75651	11.09018	Teufelsmauer	2012	16
	54.34805	10.11666	Botanical Garden Kiel	2012	16
New Zealand (exotic)	-44.16828	170.20950	Lake Pukaki East	2012	16
	-44.11396	170.12698	Lake Pukaki West	2012	15
	-43.06517	172.75104	Waipara	2012	15
	-44.59621	170.19031	Lake Aviemore West	2011	16
	-44.00175	170.47316	Lake Tekapo	2011	16
	-44.08105	170.97570	Opuha River	2011	16
	-44.18910	170.11126	Lake Pukaki South	2011	16
	-44.16550	170.21433	Hayman Road	2011	7
